# PKRxiv: A Best Practice Model for Advancing Pharmacoequity Through Open Pharmacokinetic Data Sharing

**DOI:** 10.1002/cpt.70206

**Published:** 2026-02-01

**Authors:** Shakir Atoyebi, Prajith Venkatasubramanian, Abdulafeez Akinloye, Oluwasegun Eniayewu, Brookie M. Best, Laura Else, Adeniyi Olagunju

**Affiliations:** ^1^ Department of Biochemistry, Cell and Systems Biology, Institute of Systems, Molecular and Integrative Biology University of Liverpool Liverpool UK; ^2^ Centre of Excellence for Long‐acting Therapeutics University of Liverpool Liverpool UK; ^3^ Department of Pharmaceutical Chemistry Faculty of Pharmacy, Obafemi Awolowo University Ile Ife Nigeria; ^4^ UniSA Clinical and Health Sciences, Health and Biomedical Innovation University of South Australia Adelaide South Australia Australia; ^5^ Department of Pharmaceutical and Medicinal Chemistry Faculty of Pharmaceutical Sciences, University of Ilorin Ilorin Nigeria; ^6^ Department of Pharmacy Practice and Sciences, Skaggs School of Pharmacy and Pharmaceutical Sciences University of California San Diego La Jolla California USA; ^7^ Department of Pediatrics, School of Medicine University of California San Diego La Jolla California USA; ^8^ Department of Pharmacology and Therapeutics, Institute of Systems, Molecular and Integrative Biology University of Liverpool Liverpool UK

## Abstract

Model‐informed drug development is increasingly integrated across the drug development continuum, enabling more efficient, cost‐effective, and targeted trials while reducing reliance on animal studies. Achieving pharmacoequity requires not only equitable access to medicines but also to the data and knowledge that inform drug development and regulatory decisions. To address challenges in pharmacokinetic data sharing, PKRxiv (https://pkrxiv.org/) was developed as a discipline‐specific repository designed around Findable, Accessible, Interoperable, Reusable (FAIR) principles. This tutorial introduces PKRxiv’s rationale, design, data submission and access workflows, and practical use cases. Available datasets at the end of September 2025 include over 5,500 individual drug concentration‐time data points from over 900 unique participants across 3 continents. The platform supports structured submission of pharmacokinetic, pharmacogenetic, and safety/efficacy data, with persistent digital object identifiers for discoverability and citation. Contributors can apply one of three data sharing models—unrestricted, noncommercial, or contributor‐controlled—with optional embargo periods. Users can explore datasets using the Data Explorer or Data Cards, or submit requests after providing a statement of intended use case. It enables pooling of datasets across multiple studies. Recommendations to help advance the field are proposed as data sharing becomes more widely expected: obtaining consent for unspecified future research use of data, sharing data underlying peer‐reviewed publications as standard practice, including discipline‐specific repositories in data management plans, and incentivizing post‐approval data sharing by industry. Supporting data from all therapeutic areas and population groups, PKRxiv is a critical step toward a more transparent, equitable, and collaborative future in clinical pharmacology research.

Pharmacoequity—the principle that every individual should have access to the highest quality pharmacologic care regardless of race, ethnicity, socioeconomic status, or geography—is increasingly recognized as a critical goal in clinical pharmacology.^1^ Achieving this requires not only equitable access to medicines but also equitable access to the data and knowledge that informs drug development, dosing, and regulatory decisions. However, pharmacokinetic data, which are foundational to understanding drug behavior in diverse populations, remain largely aggregated in paywalled publications and inaccessible supplementary materials. Clinical pharmacologists often share the underlying datasets through generalist repositories, which tend to function as siloed data lockers—static environments where each dataset is stored in isolation, limiting interoperability and reuse.

This fragmentation does not facilitate an ecosystem of reusable and interoperable data, and limits the potential reuse of pharmacokinetic data to inform equitable drug development.^2^ The resulting lack of transparency and accessibility disproportionately affects researchers and clinicians working outside of well‐funded academic or industry settings.^3^ It also limits the ability to conduct meta‐analyses, validate models, and optimize dosing for underrepresented populations—thereby perpetuating disparities in drug efficacy and safety.^4^, ^5^

In response to these challenges, PKRxiv (pronounced “PK archive” and available at https://pkrxiv.org/) was developed as a post‐print, managed open access, community‐driven repository for clinical pharmacokinetic data. It is a centralized, FAIR‐aligned (Findable, Accessible, Interoperable, Reusable) infrastructure for managing pharmacokinetic and pharmacogenetic data sharing following the publication of primary study outcomes in peer‐reviewed journals or preprint servers. It supports data from all therapeutic areas and across diverse population groups, ensuring broad applicability and inclusivity. Its purpose‐built design is expected to significantly enhance pharmacokinetic data sharing by lowering access barriers, fostering transparency and transforming how pharmacokinetic data are accessed and reused.

This tutorial outlines the rationale, design principles, data submission and request processes, use cases, and recommendations to realize the full potential of the platform.

## DATA SHARING CHALLENGES IN CLINICAL PHARMACOLOGY

Clinical pharmacology studies are essential for elucidating the causal relationship between drug treatment and response, thereby informing our understanding of drug safety and efficacy.^6^, ^7^ However, these studies are often resource‐intensive and their high cost has contributed to a notable scarcity of high‐quality pharmacological data, particularly in low‐ and middle‐income countries (LMICs).^6^, ^8^ This data gap exacerbates health inequities by limiting the availability of locally relevant evidence.^8^ Generating robust dose–response data typically requires adequately powered randomized controlled trials. Yet, such trials are often impractical in rare diseases where sample sizes are inherently small.^7^, ^9^ Moreover, clinical trial datasets may lack generalizability when participants are not globally representative, potentially limiting the application of evidence generated in some populations.^10^ Thus, sharing data from completed studies has been identified as a solution to advance the field, along with promotion of relevant standards for reporting and best practices for making data compliant with FAIR principles.^11^

Model‐informed drug development (MIDD) is a strategic framework that uses modeling and simulation to inform drug development decisions. It includes pharmacometrics as a core component and other modeling approaches like quantitative systems pharmacology, quantitative systems toxicology, and exposure–response modeling.^12^, ^13^ MIDD is now embedded into the regulatory framework through initiatives such as the FDA’s MIDD Paired Meeting Program and the ICH M15 guideline, with applications that include dose selection, trial design, efficacy prediction, safety evaluation, labeling decisions, and difficult‐to‐study scenarios.^14^, ^15^, ^16^ MIDD reduces reliance on animal testing, shortens development timelines (with up to 10 months saved per program), reduces costs (with $5 million savings per program), and enables smaller, more targeted clinical trials.^17^, ^18^ These benefits are achieved through the integration of diverse data sources and predictive modeling to optimize decision‐making across the drug development lifecycle.^19^

MIDD has emerged as an important discipline that integrates quantitative modeling to optimize drug therapy (e.g., in underrepresented populations). It is heavily dependent on data availability and relies on multidisciplinary collaboration, involving clinical researchers who generate the foundational data.^7^, ^18^ The quality of input data directly influences model reliability and the confidence in its application.^20^ Current publication practices often exclude raw datasets, presenting only summarized results in tables and figures.^20^, ^21^ This convention is driven by factors such as journal space constraints, dataset complexity, and concerns over data ownership.^21^ However, reliance on summary data can distort the original dataset and introduce errors into model outputs.^20^ Consequently, restrictive data‐sharing practices hinder progress in drug development and translational research.^22^

For example, population pharmacokinetic (pop‐PK) modeling requires clinical data to construct base models and assess covariate effects such as age and body weight.^23^ When sufficient data are available, inter‐ and intra‐individual variability can be accurately characterized, enabling reliable modeling of other important clinically relevant scenarios. Unfortunately, access to high‐quality clinical data remains limited for many patient groups.^24^ Pop‐PK modeling can mitigate data limitations by pooling datasets from multiple studies, but this approach is contingent on access to compatible and comprehensive data sources.^21^ The absence of centralized repositories for pharmacological data restricts the potential for robust covariate analysis and hypothesis testing.^24^ In contrast, physiologically‐based pharmacokinetic (PBPK) models often require less clinical pharmacokinetic data for initial development compared to pop‐PK models,^25^, ^26^ but high‐quality clinical data remain essential for model refinement and external validation.^25^ Inadequate validation data undermine confidence in model predictions.^27^ While published pharmacokinetic profiles can be digitized for visual predictive checks, this process is prone to error and typically limited to mean or median values.^28^ Raw clinical data are preferable, as they allow for more rigorous validation methods, such as overlaying individual data points against model prediction intervals and calculating the proportion of observations within predefined bounds. These approaches are not feasible with digitized summary data alone.^28^

Limited access to high‐quality datasets also impedes capacity‐building efforts, particularly in LMICs.^8^, ^11^ Early‐career researchers and those with less established networks face similar challenges. The scarcity of clinical pharmacology studies in LMICs further restricts opportunities for training in pharmacometrics. Some initiatives have sought to improve data access for LMIC researchers by sharing clinical trial data, but these efforts are often constrained by funding and infrastructure.^7^

Open sharing of individual‐level pharmacokinetic data is complicated by proprietary, legal, and ethical considerations.^21^ For instance, narrow patient consent may preclude future data reuse, potentially necessitating new ethics approvals.^21^, ^29^ Concerns about re‐identification are particularly acute in small studies, and proprietary claims often limit access to industry‐generated data.^21^ Researchers may also be reluctant to share data due to potential loss of publication opportunities or insufficient recognition in subsequent studies.^21^ While co‐authorship is sometimes proposed as a solution, it may not be feasible if original investigators are unfamiliar with the new analyses or unable to meet authorship criteria.^21^ In multi‐investigator studies, data sharing may require extensive internal consultation, thereby delaying access. The cost and effort required to prepare data for sharing, especially when it is not in a readily usable format, pose further barriers.^21^ Additionally, barriers to sharing scientific data through repositories include fear of misuse, reluctance to share due to previous experiences, lack of digitization, insufficient acknowledgement, absence of patient consent, and non‐binding institutional policies.^30^

## KEY DESIGN PRINCIPLES OF PKRxiv

**Figure 1:**
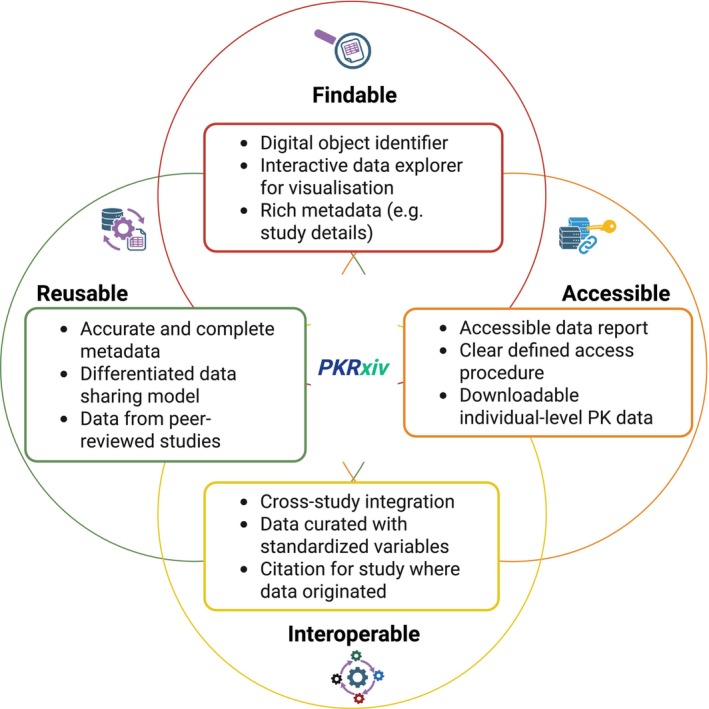
Key design principles. Specific functionalities of PKRxiv that ensure alignment with the FAIR data sharing principles (Findable, Accessible, Interoperable, and Reusable) are highlighted.

The key design principles of PKRxiv were built around the FAIR principles of research data sharing—Findability, Accessibility, Interoperability, and Reusability.^31^, ^32^ A series of consultations with key stakeholders across academia, industry, regulatory bodies, funding organizations, and IT specialists played a critical role in assessing feasibility and refining the initial concepts. Additional internal discussions with representatives from the University of Liverpool’s IT Services, Legal Department, and Research Data Team ensured alignment with institutional policies and government regulations. The outcomes of these engagements were consolidated into a comprehensive project specification document, which served as the blueprint for developing PKRxiv. Highlighted below are examples of how the functionalities align with the FAIR principles (**Figure** **1**).

### Findability

#### Digital object identifier (DOI) assignment

Once published on PKRxiv, each dataset is assigned a DOI. This transforms a dataset from a static file into a traceable, citable, and discoverable research object, boosting its visibility and accessibility across the digital research ecosystem. This persistent, globally unique link ensures the dataset can always be located even if the hosting platform changes. DOIs recognition and use by key research infrastructure (e.g., academic search engines, data repositories, citation managers, and publishing platforms) increase the likelihood of PKRxiv datasets surfacing in literature reviews, linkage in publications, and reuse in future research. DOIs also enable citation in scholarly work, improving visibility and proper attribution. Because they are standardized and machine‐readable, DOIs allow automated systems to index, retrieve, and link datasets efficiently.

#### Rich metadata

Rich metadata accompany the DOI, including study title, authors, and a permanent link to the associated data card. These make the dataset more discoverable through keyword searches.

### Accessibility

Specific functionalities in PKRxiv embody the principle of being as open as possible—ensuring unrestricted access to pharmacokinetic data—while incorporating essential governance mechanisms to uphold data quality, ethical standards, scientific integrity, and alignment with a wide range of organizational policies and data protection legislations.

#### Managed access individual‐level data

PKRxiv enables access to downloadable individual‐level pharmacokinetic data, along with associated demographic data and covariates. Only registered users can request individual‐level data on PKRxiv. Creating an account is very simple and the steps for data request using either the Data Explorer or Data Cards are clearly described in a few simple steps (https://pkrxiv.org/request/).

#### Accessible file format

Downloadable datasets are made available in CSV file format which does not require proprietary software and ensures data access regardless of technical setup or financial resources. It is both human‐ and machine‐readable, supported across virtually all operating systems and platforms for universal accessibility.

### Interoperability

Datasets are curated with standardized variables in formats, vocabularies, and ontologies (e.g., drug name, formulation, dose, and concentration) universally accepted in clinical pharmacology. This allows cross‐study integration of available datasets and dynamic linkage of pharmacokinetic datasets with available covariates (e.g., pharmacogenetics, safety and efficacy data). In addition to the adoption of standardized data formats which enhance semantic interoperability, the use of structured metadata and persistent identifiers enables seamless integration with other datasets and tools. These features allow datasets to be linked and compared meaningfully across different studies (within PKRxiv and externally) and integration with datasets from other sources for downstream analysis.

### Reusability

A core objective of PKRxiv is to facilitate pharmacokinetic data reuse by researchers outside of the original study team. In addition to making datasets downloadable in reusable file format, provision of accurate and complete metadata enables users to interpret and integrate the data appropriately. The platform employs a differentiated data sharing model, designed to accommodate the ethical approval conditions of individual studies and the data governance policies of contributing organizations—ensuring these requirements facilitate rather than hinder responsible data sharing and reuse. By curating datasets exclusively from peer‐reviewed studies, PKRxiv ensures that the data are scientifically validated, well‐documented, and reliable—thereby enhancing their reusability for future research.

**Figure 2:**
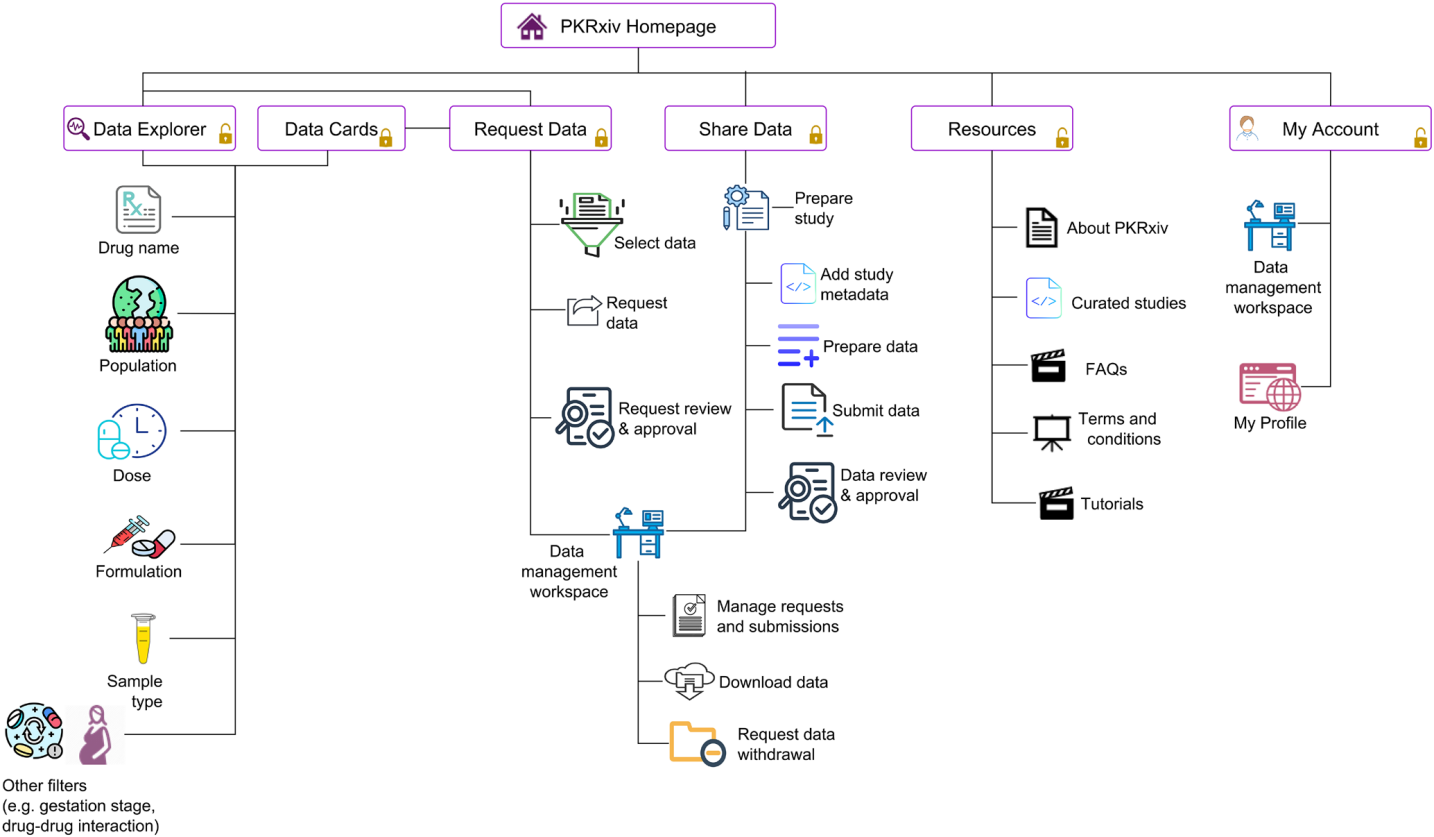
PKRxiv site map. The organization structure and navigation flow through key sections of PKRxiv guide users through data exploration, sharing, and requests. The share data page provides a brief guide on data sharing, while the request data page provides a brief guide on data requests. Access points for users include the Data Explorer and Data Cards pages. The data management workspace includes features that allow users to manage their data contributions and requests.

Here is a description of some additional design features that make PKRxiv a unique repository for pharmacokinetic data (**Figure** **2**).

### Data explorer

The PKRxiv interactive data visualization dashboard, named Data Explorer, is an openly accessible tool that allows users to visualize available datasets. Data could be explored at four different levels: concentration‐time profile, pharmacokinetic parameters (e.g., minimum and maximum concentration), demographic data, and data report. Using the robust collection of interactive filters, the dynamic tool displays the concentration‐time profile, pharmacokinetic parameters, and aggregated participants’ demographic data. These are computed in real‐time from all available datasets as the filters are selected. Filters include drug name, dose, study design, sample type, route of administration, and formulation, allowing detailed dataset exploration for use case suitability assessment. All users can use the Data Explorer to generate a downloadable data report that includes: (i) selected filters; (ii) aggregated participants’ demographic data; (iii) concentration‐time plots; (iv) aggregated pharmacokinetic parameters.

### Data cards

**Figure 3:**
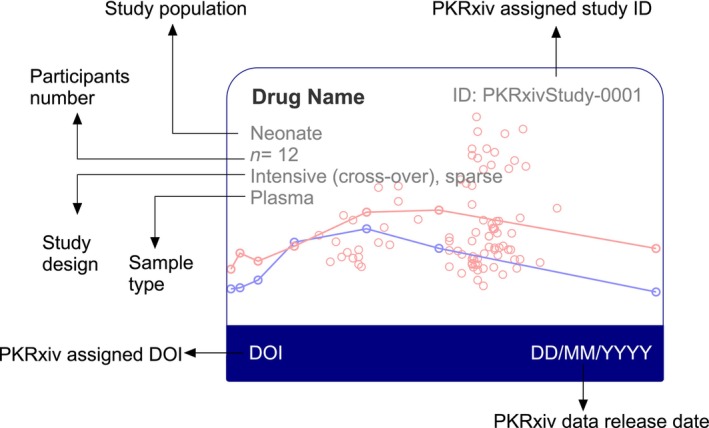
Data card. Some of the attributes of the study that generated the dataset are indicated on the data card. The data card features: the study drug, the study population, the number of study participants, the study design, the sample matrix, the date of publication of the dataset on PKRxiv, the internal identifier number for the dataset, and a digital object identifier assigned to the dataset.

Data Cards for five most recently curated datasets are displayed on the home page. The Data Cards page, accessible to registered users, offers a structured and interactive environment for accessing curated datasets. The unique design of PKRxiv Data Cards (**Figure** **3**) features essential metadata, including drug name, study population, number of participants, study design, sample type, data curation date, and persistent DOI link. Simple visual inspection enables users to identify whether datasets are sparse, intensive, or a combination of both. The sidebar of the Data Cards page features a collection of filters like the Data Explorer, allowing customized and efficient dataset exploration and retrieval. By standardizing the presentation of study metadata and integrating source traceability, the Data Cards interface promotes rigorous data examination and improves transparency.

### Differentiated data sharing model

To accommodate the diverse needs of contributors and users, PKRxiv implements a differentiated data sharing model framework that aligns with international standards for ethical and legal data governance. Under this framework, data contributors are required to apply one of three distinct models to each dataset shared through PKRxiv.

#### Unrestricted

Datasets shared under this model are openly accessible to all users under the general PKRxiv terms and conditions. These typically include individualized, anonymized datasets and associated covariates, enabling broad reuse in secondary analyses, model validation, and educational applications. This approach aligns with the FAIR principles and promotes open science practices. Requests for data are approved by the PKRxiv team.

#### Non‐commercial

Access is limited to non‐commercial research purposes. Use cases involving commercial interests, such as proprietary drug development or monetized analytics, are excluded. This model supports academic and public‐sector research in situations where the sponsor’s policy requires that data should be as open as possible, as closed as necessary to safeguarding intellectual property and ethical boundaries (e.g., Horizon Europe Open Science Policy^33^). Requests for data shared under this model are subject to the general PKRxiv terms and conditions and approved by the PKRxiv team.

#### Contributor‐controlled

In this model, data access decisions are delegated to the original contributor, who determines the terms of sharing and the applicable data use agreement. This will usually be the member of the study team who deposited the dataset on PKRxiv (e.g., senior author or a co‐author with necessary authority). This approach reflects evolving norms in participant‐level data governance, where contributors retain control over sensitive or proprietary datasets. The PKRxiv team is unable to approve or reject requests under this model.

These models are implemented in accordance with existing data governance policies and based on identified needs of stakeholders. By offering structured, tiered access, PKRxiv removes barriers to data sharing and facilitates responsible data reuse across multiple levels while ensuring compliance with ethical and legal standards.

## USER ACCOUNT CREATION

PKRxiv operates a tiered user level which includes Admin Users, Data Curators, Regular Users, and Ordinary Users. Only Admin Users and Data Curators have access to the backend. Regular Users have access to data submission and request functionalities, while Ordinary Users do not until they create a secure password and accept the platform’s terms and conditions. The latter tier only applies to the in‐house data curation process where data contributors have requested support. User account creation is initiated by the PKRxiv team, and the user will receive an email prompting them to set up a secure password and to confirm acceptance of terms and conditions, after which they become Regular Users.

To access and contribute to the PKRxiv platform, users are required to complete a registration process that captures essential identity and affiliation details. Specifically, registrants must provide their full name, institutional affiliation, professional title, email address, and establish a secure password. As part of the onboarding process, users must also formally accept PKRxiv’s standard data sharing terms and conditions, which govern the ethical sharing and reuse of datasets. Upon successful registration, users receive a confirmation email verifying account activation and granting access to the platform’s data submission and request features. This process ensures accountability, traceability, and compliance with data governance standards.

## HOW TO SHARE DATA ON PKRxiv

**Figure 4:**
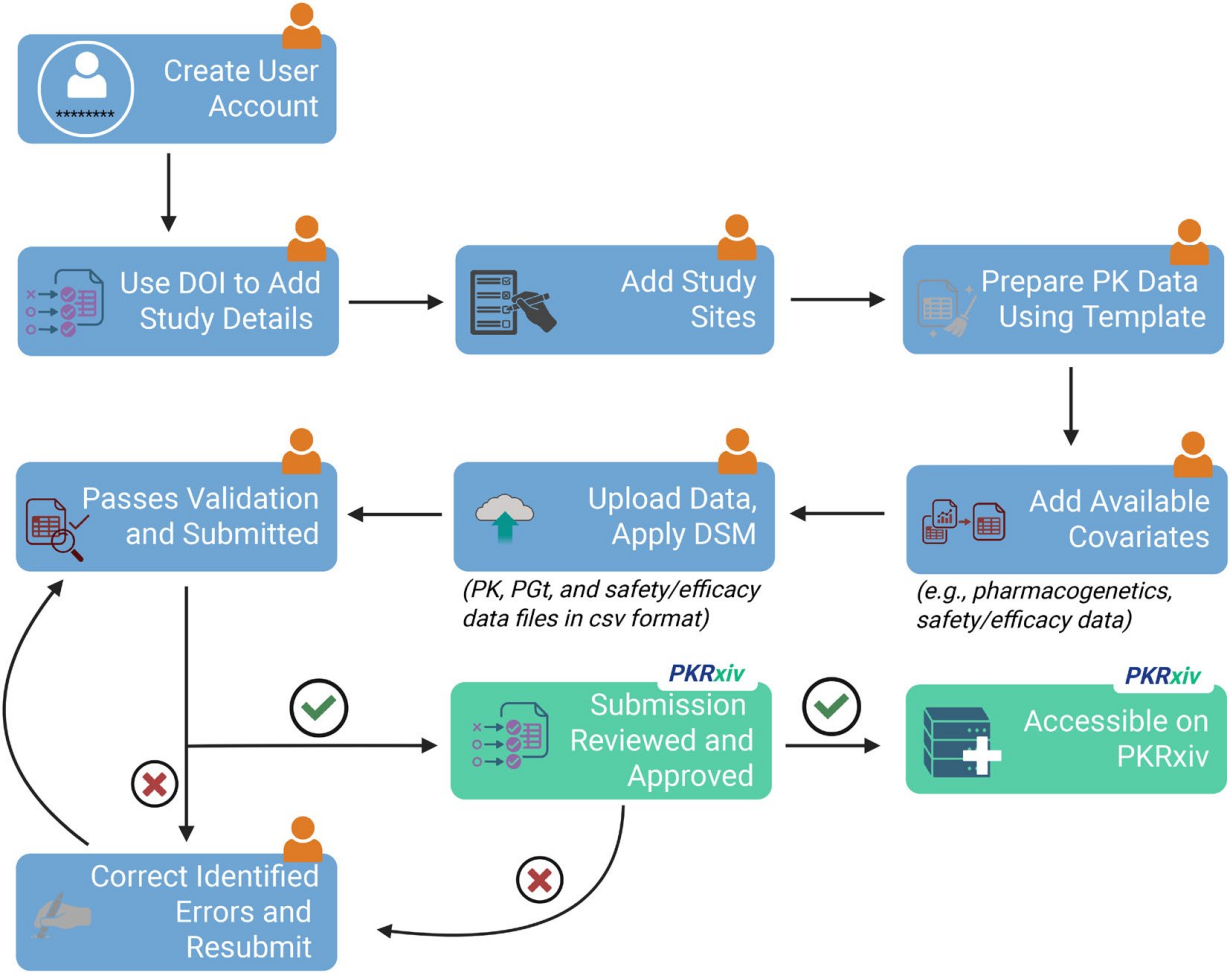
PKRxiv data sharing workflow. The process begins with the addition of the study details and study sites. The data contributor should prepare the data using the data template available on PKRxiv. Once the data are prepared and uploaded, the data sharing model preferred by the contributor can be applied. Upon successful review and approval, the dataset is assigned a unique DOI and published. The orange person icon represents the data contributor. csv—comma separated values, DOI—digital object identifier, DSM—data sharing model, PGt– pharmacogenetic, and PK—pharmacokinetic.

Sharing data through PKRxiv involves a few simple steps (**Figure** **4**).

### Step 1—Add study details

After logging in, navigate to the Data Management Workspace under My Account. Click the “Create New Submission.” This is also accessible on the Share Data page. Here, provide the DOI of the peer‐reviewed article in which the primary outcomes for the study were published. Clicking on “Verify URL and populate fields” creates a data submission form populated with study details like title, authors, and affiliation, journal, and publication date. Affiliations are sometimes not properly indexed, leading to incomplete abstraction, and data contributors may need to verify this field and manually enter any missing details (e.g., country location).

### Step 2—Add study sites

Manually enter all the clinical study sites as listed in the published article, including site name, city and country location.

### Step 3—Prepare data files

Use the PKRxiv data curation template to prepare data for submission. This structured workbook contains three spreadsheets: one for pharmacokinetic data, one for pharmacogenetic data, and one for safety and efficacy data.

- Pharmacokinetic data file: It contains 27 columns (**Table** **1**), including 21 required data fields (e.g., study DOI, drug name, sample type, drug concentration, study population, anonymized participant ID) and six optional fields such as gestational age if pregnancy study, postpartum age if breastfeeding study. Validation rules embedded within the spreadsheet prevent modification of predefined columns.
- Pharmacogenetic data file: This includes two required data fields (study DOI and participant ID) for linkage to pharmacokinetic data, and unspecified number of columns. Replace placeholders named SNP_descriptors with study‐specific pharmacogenetic data.
- Safety and efficacy data file: This also includes two required data fields (study DOI and participant ID) for linkage to pharmacokinetic data, and an unspecified number of additional columns. Replace placeholders named safety_descriptor and date_safety_descriptor with study‐specific safety and efficacy data.
- Save each file in CSV format.

**Table 1:** List of variables in PKRxiv data curation templates.

Variable name	Template	Description
admin_route	PK	The route of drug administration for the study participant
age	PK	Age of the study participant at the time of sample collection
age_unit	PK	Unit of measure for the age of the study participant
body_weight_kg	PK	Body‐weight of the study participant at the time of sample collection in kilograms
co_administered_agent_name	PK	Name(s) of drugs co‐administered with the study drug especially in drug–drug interaction studies
concentration	PK	Measured drug concentration after the study sample was analyzed
concentration_unit	PK	Unit of measure for the drug concentration
efficacy_descriptor	PD	Descriptor for the observed efficacy of the study drug
date_efficacy_descriptor	PD	Date the efficacy descriptor was observed (recorded)
date_safety_descriptor	PD	Date the safety descriptor was observed (recorded)
dose_unit	PK	Unit of measure for dose administered to the study participant
dosing_schedule	PK	Schedule of dosing of the drug for the study participant
drug_name	PK	Name of the study drug
duration_on_drug_days	PK	Length of time (in days) that the study participant has been taking the drug before the time the study sample was taken
formulation_type	PK	Type of formulation of the drug administered to the study participant
gender	PK	Gender of the study participants
gestation_age_in_weeks	PK	(For pregnancy‐postpartum studies) gestational age in weeks at the time of sample collection from a pregnant study participant
gestation_stage	PK	(For pregnancy‐postpartum studies) the period in pregnancy or after pregnancy at the time of sample collection
initial_dose	PK	The amount of dose administered to the study participant
participant_study_id	PK, PD, PGx	The anonymized identifier assigned to the participant in the study
pk_sample_collection_date	PK	The date of collection of the study sample
population_type	PK	Population(s) the pharmacological data was drawn from
postpartum_age_in_days	PK	(For pregnancy‐postpartum studies) the number of weeks after delivery at the time of sample collection
race	PK	Race recorded of study participant
safety_descriptor	PD	Descriptor for the observed safety of the study drug
sample_type	PK	Type of matrix the study sample was withdrawn from (e.g., blood, breastmilk)
SNP_descriptor	PGx	Description of the single nucleotide polymorphism in the study participant
study_design	PK	Type of design of pharmacokinetic study (e.g., crossover or intensive)
study_details	PK	Classification of the study type (e.g., basic pk, food‐effect)
study_doi	PK, PD, PGx	Digital object identifier assigned to the published study by the publisher
time_after_dose	PK	Time of sampling after the dose administration
time_after_dose_unit	PK	Unit of measure for the time of sampling after the dose administration

PD, pharmacodynamic; PGx, pharmacogenetics; PK, pharmacokinetics.

### Step 4—Upload data files and apply data sharing model

To upload the pharmacokinetic data file, click the “Choose file” button to navigate to its location on your computer. To add associated pharmacogenetic data and/or safety and efficacy data, tick the relevant box to open the data upload function. In each case, click the “Choose file” button and navigate to its location on your computer.

### Optional embargo period

Users have the option to apply an embargo period at this stage by indicating a preferred date for their dataset to become available for request by PKRxiv users. This may be necessary to allow relevant publication of ongoing additional analysis on the dataset by the investigators.

### Step 5—Validate and submit

Click on the submit button to validate and submit dataset for review. PKRxiv has built‐in functionalities to validate the dataset and identify any errors (e.g., missing compulsory fields, presence of unidentifiable data column, upload of wrong file format, etc.). A detailed validation report lists out all identified errors on the page to allow users to locate and correct them.

### Step 6—Manage contribution

Once submitted, you can manage your submission under My Contributions tab within the Data Management Workspace. Each submission is displayed along with its status (awaiting approval, approved (published), approved (embargoed), or rejected), applied data sharing model, date and time shared on PKRxiv, and details entered on the data submission form. The data management workspace also has provision for users to request deletion of their dataset, if necessary, for instance due to identified major errors.

If you have data for multiple studies, each study must be contributed separately using the study publication DOI as described above.

## HOW TO REQUEST DATA ON PKRxiv

**Figure 5:**
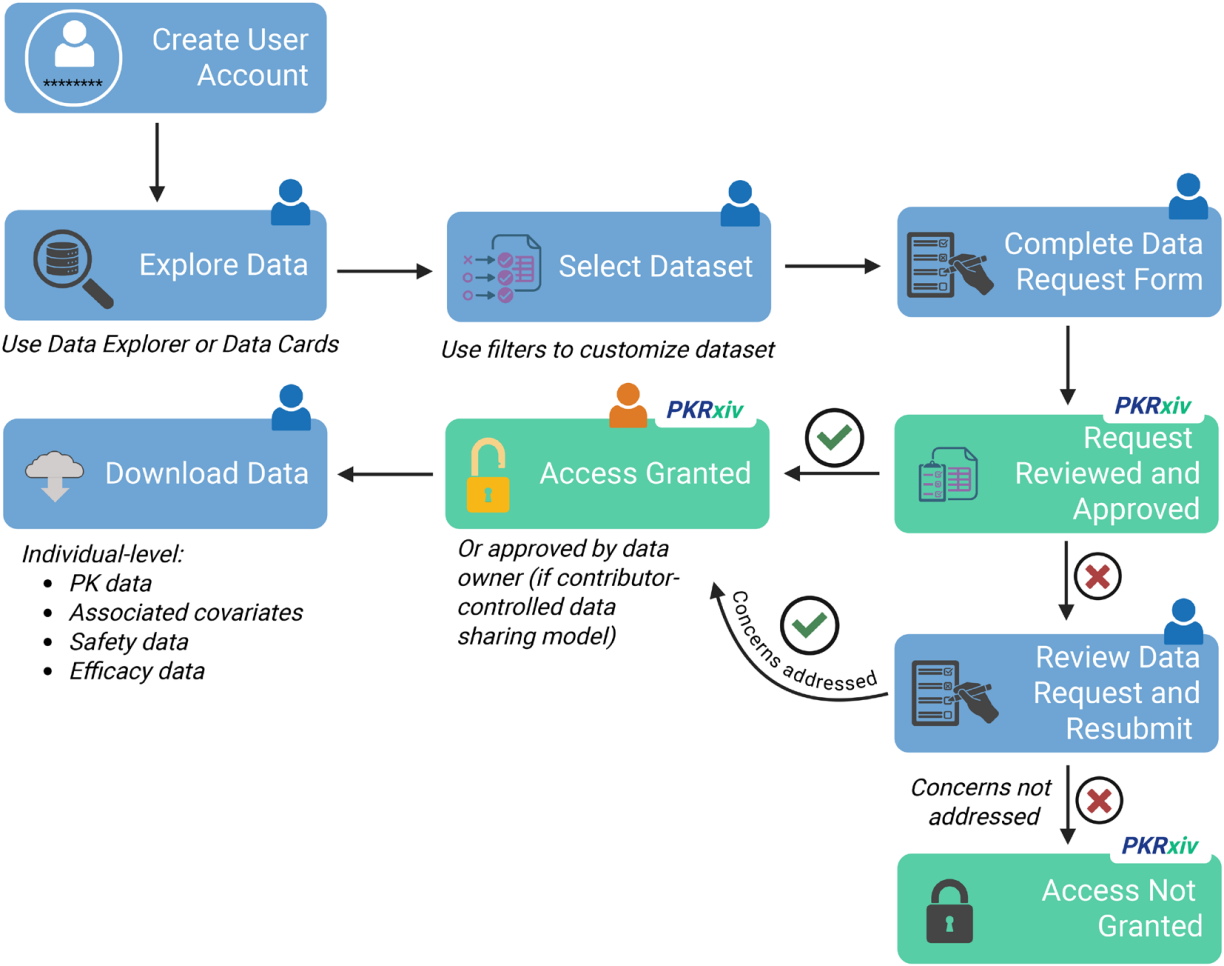
PKRxiv data request workflow. The process begins with dataset exploration using the Data Explorer or Data Cards. Once the user selects datasets and completes the data request form (including a statement of use case), the submitted request is reviewed (by the PKRxiv team or data contributor). Upon successful review, secure access is granted to anonymized individual‐level pharmacokinetic data along with associated covariates. The blue and orange person icons represent the data requester and the data owner, respectively.

PKRxiv offers two distinct pathways for data request: the Data Explorer and the Data Cards (**Figure** **5**). Key components of these unique features were described above. Like data sharing, only Regular Users have access to the data request section.

### Step 1—Explore data

Exploring available datasets using the collection of filters in the Data Explorer tool or the Data Cards page allows users to understand what is available, pool datasets across multiple studies based on specific criteria, and assess suitability for their intended use case. Importantly, while unregistered users can use the Data Explorer tool, only registered users have access to the Data Cards page.

### Step 2—Select dataset

To select datasets for request based on selected filters, navigate to the Data Request tab of the Data Explorer and click on “Request Selected Dataset.” Alternatively, you can navigate to the Data Cards page and click on the “Request this dataset” button to select a dataset from a single study. To pool datasets from multiple studies, use the relevant filters and click on the “Request Datasets” at the top of the filters.

### Step 3—Complete data request form

A data request form, pre‐populated with details of dataset(s) selected in Step 2 is generated for completion. Here, provide a very clear statement (up to 1,000 characters) describing the intended use case for the requested data (e.g., type of model, project funder, your collaborators, any commercial interest, and your intention to publish output). Complete the form by accepting data sharing terms and conditions.

### Step 4—Submit and manage request

Once submitted, you can manage your request under My Data Requests tab within the Data Management Workspace. Each request is displayed along with its status (awaiting approval, approved, or rejected), request date and time, details provided in the data request form, one‐time download link to approved datasets in CSV format, and Full citation(s) for data reuse.

### Data sharing and requests under contributor‐controlled data sharing model

In addition to the steps highlighted above, sharing datasets under the contributor‐controlled data sharing model requires that the contributor provides a data sharing agreement at the time of data submission. A dialog box to upload this becomes available whenever this model is selected. The platform stores this agreement along with the dataset. Whenever a dataset in this category is requested, the platform generates an agreement for the requester to complete and upload as part of the process. Upon submission, the contributor receives an email notification asking them to respond to the request within their Data Management Workspace.

In addition to My Contributions and My Data Requests tabs, the Data Management Workspace also includes a third tab named Requests for My Data. This is designed for managing requests for datasets to which the contributor‐controlled data sharing model has been applied. Here data contributors will see all requests for their datasets and the status of each request. After reviewing the request, intended use case, and the completed data sharing agreement, the data contributor can approve (if satisfactory) or reject the request (option to provide reason).

Only data contributors have access to the data request approval functionality within the Data Management Workspace and retain exclusive right to manage data access. PKRxiv automatically sends reminder emails to contributors if requests remain unaddressed after a few days.

## PKRxiv STATUS AND EARLY USE CASES

Datasets publicly available for request on PKRxiv at the end of September 2025 include over 5,500 individual drug concentration‐time data points from six biological matrices collected from more than 900 unique participants who participated in clinical studies conducted in locations across Africa, Asia, and North America. A summary of key attributes of each dataset is presented in **Table** **2**. Some of the datasets include associated covariates like pharmacogenetics and efficacy data. Population groups represented include adults, neonates, infants, pregnant, and postpartum women.

**Table 2:** Some characteristics of deposited pharmacological datasets hosted on PKRxiv.

Study	Drug/Indication	Study design	Location	Population	Sample type	Data Points/Participants	Associated datasets	
							PGx	Efficacy/Safety
Olagunju *et al*. (2015a)^37^	Efavirenz/HIV	Sparse/Intensive (parallel)	Nigeria	Pregnant/postpartum	Plasma (Blood)	589/*n* = 182	Yes	Yes
Olagunju *et al*. (2015b)^50^	Efavirenz/HIV	Sparse/Intensive	Nigeria	Pregnant/postpartum/ infant	Plasma (Blood), breastmilk, dried milk spot	812/*n* = 127	Yes	Yes
Akinloye *et al*. (2024)^51^	Nitazoxanide (Tizoxanide)/ Anti‐protozoan	Intensive (Cross‐over)	Nigeria	Adult	Plasma (Blood)	288/*n* = 17	No	No
Akinloye *et al*. (2022)^52^	Dolutegravir/HIV	Intensive	Nigeria	Pregnant/postpartum	Dried blood spot	80/*n* = 10	No	No
Olagunju *et al*. (2016a)^53^	Nevirapine/HIV	Sparse/Intensive (parallel)	Nigeria	Pregnant/postpartum/ infant	Plasma (Blood), dried milk spot	692/*n* = 120	Yes	Yes
Olagunju *et al*. (2016b)^54^	Nevirapine/HIV‐1	Sparse/Intensive (parallel)	Nigeria	Pregnant/postpartum	Plasma (Blood)	570/*n* = 218	Yes	Yes
Waitt *et al*. (2019)^55^	Dolutegravir/HIV‐1	Intensive (Cross‐over)	Uganda, South Africa	Pregnant/postpartum/neonates	Plasma (Blood), cord blood	673/*n* = 28	No	No
Mulligan *et al*. (2018)^56^	Dolutegravir/HIV‐1	Intensive	USA	Pregnant/postpartum/neonates	Plasma (Blood)	527/*n* = 29	No	No
Eniayewu *et al*. (2024)^39^	Efavirenz/HIV‐1	Sparse/Intensive	Nigeria	Pregnant/postpartum	Cervicovaginal (swab), Plasma (Blood)	329/*n* = 109	Yes	Yes
Cressey *et al*. (2012)^35^	Efavirenz/HIV‐1	Intensive	USA, Thailand	Pregnant/postpartum	Plasma (Blood)	400/*n* = 25	No	No
Tran *et al*. (2016)^57^	Rilpivirine/HIV‐1	Intensive	USA	Pregnant	Plasma (Blood)	589/*n* = 31	No	No

ML, machine learning; PBPK, physiologically‐based pharmacokinetic; PGx, pharmacogenetics; PK‐PD, pharmacokinetic‐pharmacodynamic; pop‐PK, population pharmacokinetic.

Since the launch of PKRxiv in January 2025, several requests by users from multiple countries have been approved. Stated use cases at the point of request can broadly be grouped as follows:

- Pharmacokinetic model validation.
- Machine learning model development and validation
- Comparison of pharmacokinetic performance of models developed on different platforms.
- Evaluation of variability in drug response between publicly available pharmacokinetic datasets and clinical data obtained from a hospital cohort.
- Comparison of plasma concentration‐time data from plot digitization with individual‐level data.

### Demonstration of a use case example

This is based on a revalidation of a previously described pregnancy model for the antiretroviral drug efavirenz,^34^ initially validated with digitized plasma concentration‐time data from the study by Cressey *et al*.^35^ In brief, a total of 100 virtual females receiving 600 mg efavirenz once daily in the third trimester of pregnancy were simulated. Concentration‐time data were obtained at steady state and used to compute the area under the concentration‐time curve.

**Figure 6:**
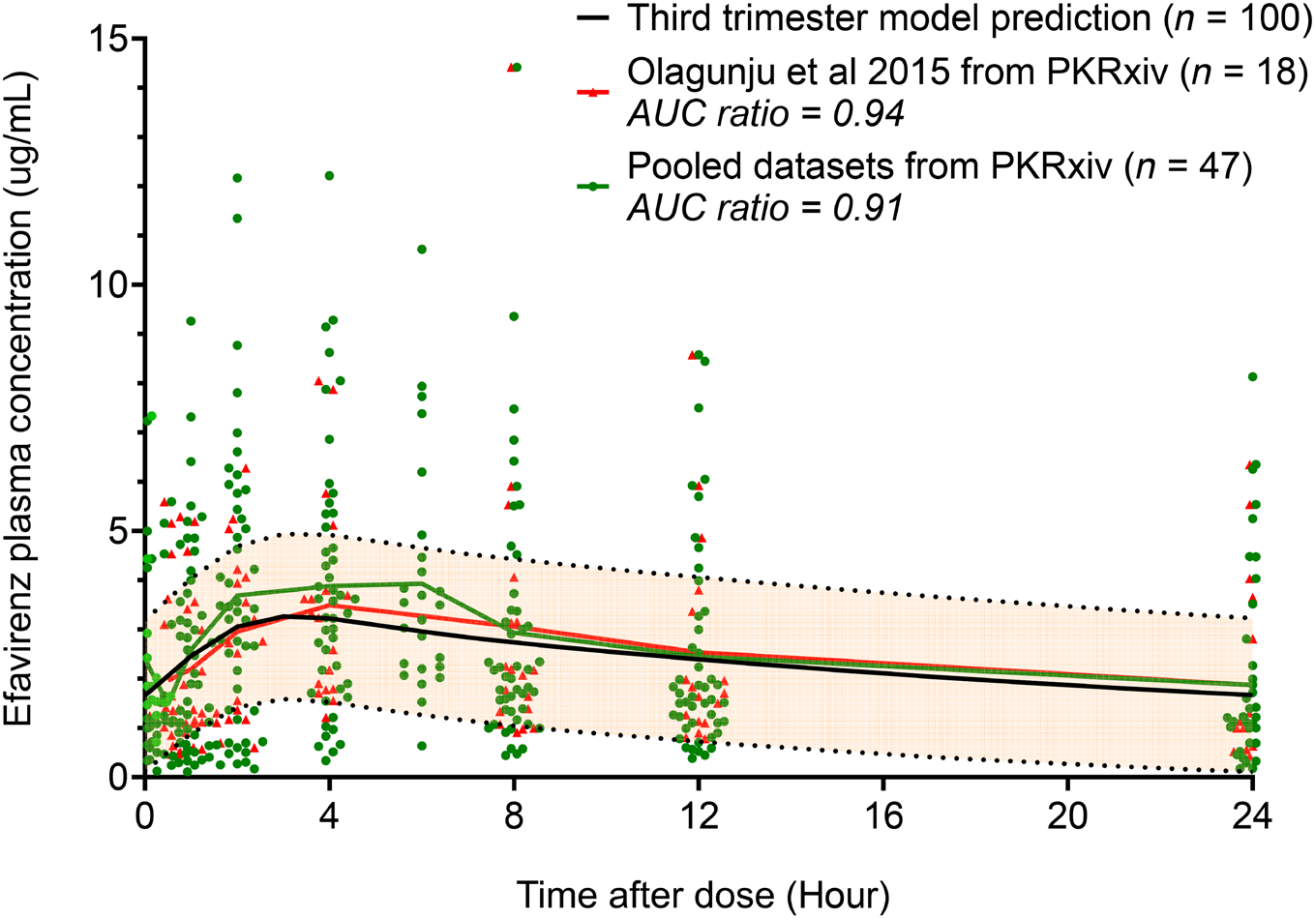
Revalidation of a pregnancy PBPK model of 600 mg orally administered efavirenz using individual‐level datasets from PKRxiv. Validation with one dataset (Olagunju *et al*.; 126 third trimester plasma concentration data points from 18 participants)^37^ resulted in good model prediction (black), with predicted/observed AUC ratio of 0.94, and the majority of data points (shown in red) within the prediction window. Predictions were also compared with pooled datasets from three studies available on PKRxiv at the time of this analysis (shown in green; 353 third trimester plasma concentration data points from 47 participants), AUC ratio of 0.91. Data are presented as mean with standard deviation or individual replicates.

- Data acquisition from PKRxiv: To request individual‐level pharmacokinetic datasets for validation from PKRxiv, the following combination of filters was applied on the Data Cards page: Efavirenz, Plasma (Blood), Pregnant/Postpartum, Oral, Tablet, 600 mg, All Study Designs, Multiple‐dose (steady state), Basic pharmacokinetics, and Third trimester. The Data Request Form was completed and submitted. Upon approval, an email was received and the data access link within the “Data Management Workspace” under “My Account” was used to download the datasets. At the time of this analysis, the downloaded CVS file included three datasets from published studies that matched the filters described above:
  ◦ Cressey, T. R. *et al*. (2025). Efavirenz pharmacokinetics during the third trimester of pregnancy and postpartum (dataset). PKRxiv, University of Liverpool. https://doi.org/10.58033/QXYN‐NX97.^35^, ^36^
  ◦ Olagunju, A. *et al*. (2025). Pharmacogenetics of pregnancy‐induced changes in efavirenz pharmacokinetics (dataset). PKRxiv, University of Liverpool. https://doi.org/10.58033/NMMY‐PR11.^37^, ^38^
  ◦ Eniayewu, O. *et al*. (2025). Pharmacogenetics of efavirenz exposure in cervicovaginal fluid during pregnancy and postpartum (dataset). PKRxiv, University of Liverpool. https://doi.org/10.58033/G748‐JP07.^39^, ^40^
- Model revalidation summary (**Figure** **6**): In brief, the model was first revalidated using one dataset (Olagunju *et al*.) which included 126 third trimester plasma concentration data points from 18 participants.^37^ The mean predicted/observed area under the concentration‐time curve (AUC) ratio was 0.94, with most data points falling within the prediction window. When validation was extended to all three datasets (totaling 353 third trimester plasma concentration data points from 47 participants), the AUC ratio was 0.91. Only intensive pharmacokinetic data were used for the purpose of this demonstration.

Availability of individual‐level pharmacokinetic data allows for the estimation of the proportion of observed data points captured within the prediction window—a useful semi‐quantitative metric for assessing model performance.

## ANTICIPATED LONG‐TERM OUTCOMES

As individual‐level pharmacokinetic data and associated covariates are the cornerstone of MIDD, which is critical for model development and validation, access to such data through PKRxiv is expected to contribute to further progress in this important area. When granular datasets from clinical pharmacology studies are shared openly and in standardized formats, they can be reused for:

- Optimizing drug development by informing dose selection, trial design, and population‐specific adjustments.
- Validating and refining models, ensuring they perform reliably across different demographic and clinical contexts.^21^
- Improving global representativeness of data used in MIDD, especially when data from underrepresented populations—such as children, pregnant women, elderly individuals, and patients from LMICs—are included.^11^
- Improving regulatory science by enabling external validation of models used to generate data included in regulatory submissions
- Meta‐analysis and model‐based meta‐analysis, which can uncover patterns and gaps in existing knowledge.^21^
- Educational purposes, allowing students and early‐career scientists to engage with real‐world data and develop modeling skills.^21^
- Promoting data transparency, which will improve research reproducibility and build confidence in findings from clinical pharmacology studies.^20^
- Increased research efficiency as fewer resources are devoted to duplicating research when the required data are available and accessible from a previous study.^21^

PKRxiv was specifically designed to enable these outcomes, providing the needed infrastructure for managing data sharing in line with FAIR principles to support data‐driven drug research and development. For PKRxiv, some of these outcomes (e.g. meta‐analyses, enabling external validation of models in regulatory submissions) will become feasible only once a critical mass of data is uploaded to the repository since models are often validated for one molecule at a time. Importantly, the Organization for Economic Co‐operation and Development (OECD) guidance on PBPK model validation recommends a read‐across approach in the absence of in vivo kinetic data. This approach involves establishing confidence in the predictive ability of a model using chemical analogues for which in vivo kinetic data are available.^41^ The possibility of placing an embargo on deposited data could encourage researchers to contribute data early as they are able to stipulate when the data would be widely available. This approach could give them sufficient time to exhaust their intended study‐specific use of the data before the dataset becomes widely available to others.

## RECOMMENDATIONS

### Obtain consent for future unspecified research use of data

In clinical pharmacology studies, include provision for future unspecified research use of fully anonymized data in participant information leaflet and consent form. This ensures that potential ethical constraints are avoided after study completion. This is especially important because major research funders now require datasets generated from funded projects to be shared to promote transparency, reproducibility, and public benefit. Here are a few examples:

- Data from all research funded by US *National Institutes of Health (NIH)* are expected to be shared as early as possible, no later than the time of publication or the end of the project period, whichever comes first.^42^
- *Wellcome* expects researchers to share data, software, and materials openly with minimal restrictions, ideally at the time of publication, using suitable repositories.^43^
- The *Gates Foundation* mandates immediate open access to all data supporting published research, including metadata, to promote transparency and reproducibility.^44^
- The UK *National Institute for Health and Care Research* supports open data sharing to maximize public benefit, requiring data management plans and repository use, while respecting ethical and legal constraints.^45^
- The *European Commission* mandates open access to research data under its Horizon Europe programme, requiring outputs to follow FAIR principles and be deposited in trusted repositories, unless justified restrictions apply.^46^
- The *European and Developing Countries Clinical Trials Partnership* encourages data sharing from funded clinical trials, especially during public health emergencies, to maximize research transparency and impact.^47^

### Share data underlying results published in peer‐reviewed journals

An increasing number of academic journals now require authors to share the data underlying their published results in publicly accessible repositories. This shift reflects a broader movement toward transparency, reproducibility, and adherence to FAIR data principles. Some major publishers have implemented or strengthened their data sharing mandates, often requiring data availability statements and repository deposition as conditions for publication. PKRxiv is provisioned with functionalities to support these efforts, including DOI assignment. This allows data citation for new data shared alongside the publication or secondary data used for the analysis. Authors can reference PKRxiv in their data availability statements, specifying the repository location and any access conditions based on the applied data sharing model. Since the underlying publication DOI is required as part of the submission process, authors can deposit their manuscript in a preprint server and use the associated DOI to deposit data in PKRxiv. This aligns with journal expectations for transparency and reproducibility, ensuring that readers and reviewers can locate and evaluate the underlying data supporting published results. While PKRxiv does not currently support unpublished phase 1 clinical trial data (e.g., reported on clinicaltrials.gov), there is potential to add this functionality as part of a future upgrade if there is a clear need in the field.

### Use discipline‐specific repositories for pharmacokinetic data

To facilitate access to and reuse of pharmacokinetic data, researchers are encouraged to include the use of discipline‐specific repositories like PKRxiv in their data management and sharing plan. Its functionalities are tailored to the unique formats of concentration‐time data, metadata standards, and visualization requirements in this field, facilitating data discoverability and integration across studies. Recognizing that generalist repositories may not always be suitable for domain‐specific data, NIH explicitly recommends the use of discipline‐appropriate repositories. A recent study by Miao *et al*.^48^ revealed that only 2% of pharmacogenomic publications are publicly accessible, and even those often lack straightforward mechanisms for requesting the underlying data. PKRxiv supports the curation of covariates data like pharmacogenetics, as well as safety and efficacy which are essential for secondary analyses and MIDD.

### Incentivize industry to share data in accessible repository after drug approval

To enhance transparency and scientific value, pharmaceutical companies should be incentivized to deposit clinical and pharmacokinetic data in accessible repositories following drug approval. In a review of the 2013 data sharing commitments by major players in the pharmaceutical industry, the following two updates were recommended: (1) participant‐level data from clinical trials that supported drug approvals should be shared, irrespective of continuing follow‐up; and (2) data from clinical trials not directly supporting drug approvals should be shared within a clearly defined timeframe of study completion or publication.^49^ The contributor‐controlled data sharing model in PKRxiv was specifically designed to align with the often complex mix of considerations in industry‐sponsored studies.

## FUNDING

This work was supported by the Wellcome Trust (227288/Z/23/Z). For the purpose of open access, the authors have applied a CC BY public copyright license to any Author Accepted Manuscript version arising from this submission.

## CONFLICTS OF INTEREST

The authors declared no competing interests for this work.
